# Supplementary material for: Observation of the ponderomotive effect in non-valence bound states of polyatomic molecular anions
Source: Nat Commun. 2021 Dec 7;12:7098. doi: 10.1038/s41467-021-27468-1 (PMC8651741; doi:10.1038/s41467-021-27468-1)
Supplement: Supplementary file 1 — Supplementary Information [file 41467_2021_27468_MOESM1_ESM.pdf]

Supplementary Information for

Observation of the Ponderomotive Effect in Non-Valence Bound States of Polyatomic Molecular Anions

Do Hyung Kang[1], Jinwoo Kim[1], Heung-Ryoul Noh[2], and Sang Kyu Kim[1]*

[1]*Department of Chemistry, KAIST, Daejeon 34141, Republic of Korea*

[2]*Department of Physics, Chonnam National University, Gwangju 61186, Republic of Korea*

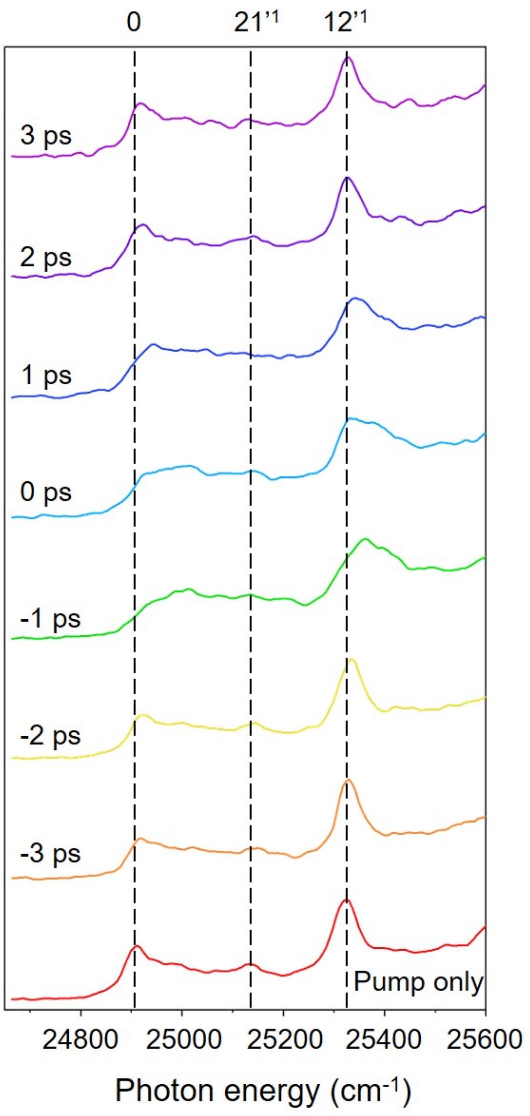

Supplementary Fig. 1 Photodetachment spectra of 4-CP QBS in various pump-control pulse delay at ~10 GW/cm² pump laser intensity. Source data are provided as a Source Data file.

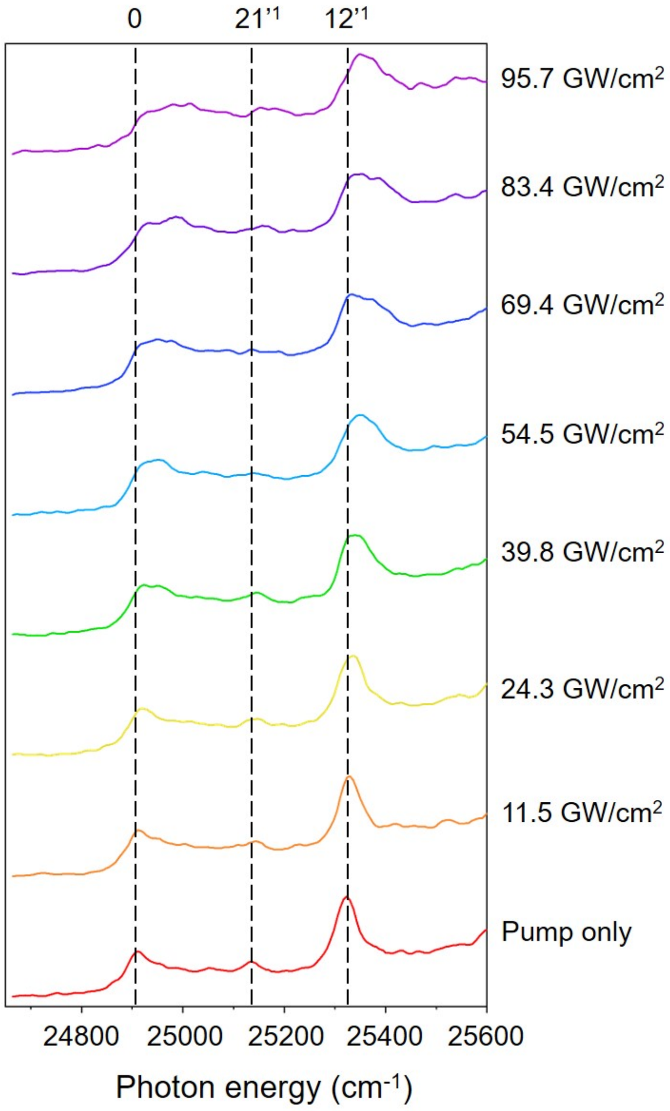

Supplementary Fig. 2 Photodetachment spectra of 4-CP QBS in various control pulse intensity at ~10 GW/cm^2 pump pulse intensity. Source data are provided as a Source Data file.

Supplementary Note 1

Details of the subtraction of direct detachment in photodetachment spectra

In the photodetachment spectrum, the direct detachment by one-photon transition reveals stepwise feature at electron affinity (EA) as seen in Fig. 1. On the other hands, the stepwise feature from the direct detachment also could be revealed near the most FC-active vibrational state of the DBS or QBS[1]. Since the direct detachment cross-section usually follows the FC-factor, stepwise feature could be revealed when the excitation pulse exceeds the energy of the most FC-active vibrational mode in neutral electronic ground state.

Because of the loosely bound nature of the DB- or QB-electron, the geometry of the DB or QB is barely differed from the neutral ground state geometry[2]. Those makes the stepwise feature located to the blue-side of the most FC-active DBS vibrational peak, shifted with the almost same amount of that of EA. For the DBS or QBS with low binding energies, the vibrational peak of DBS or QBS could be overlapped to the stepwise direct detachment. This makes the vibrational peak to be more asymmetric to the blue side, consequently makes hard to exact estimation of the extent of ponderomotive shift and broadening. For the 4-CP$^-$ QBS and 4-BP$^-$ DBS (to be referred below), which have small electron binding energy similar to the picosecond bandwidth (~20 cm^{-1}), the most FC-active vibrational peaks have quite asymmetric peak shapes because of aforementioned stepwise feature overlapped to the vibrational peak. We carefully eliminate this feature by fitting in order to evaluate the extent of the ponderomotive shift more precisely. We used error-function to represent the stepwise direct detachment;

$$\mathrm{erf}(z) = \frac{2}{\sqrt{\pi}} \int_0^z e^{-t^2} dt \qquad \text{Eq. S1}$$

To be specific, the direct detachment cross-section is usually expressed by the Wigner's threshold law, which is proportional to the $\sqrt{\mathrm{eKE}}$. However, the Wigner's threshold law could not represent the vibrational aspect of the direct detachment cross-section as mentioned above, rather usually being adopted to the 'electronic' detachment cross-section. In the very narrow energetic range compared to the 'electronic' detachment cross-section, error-function could

be reasonable to represent the stepwise feature originated from the vibration.

Supplementary Fig. 3 (a) is the extracting procedure of the 4-CP$^-$ QBS 12′^{1} vibrational peak from the direct detachment. We subtracted error function in two points: EA and EA+E(v_{12}). This eliminates the stepwise direct detachment features at the EA and the most FC-active vibrational state. Resultant photodetachment spectra are described in Supplementary Fig. 3 (b). Note that the pump only spectrum has quite symmetric structure after the subtraction of the direct detachment, and the shift and broadening are more clearly observable when the various control pulse intensity was used (Supplementary Fig. 3 (c) and (d)). Note that we also corrected the position of the error functions as amount of the full-ponderomotive shift (free electron ponderomotive shift) when the control pulse was used (Supplementary Fig. 3 (c)), because the detachment threshold would be shifted as same as full-ponderomotive shift. Supplementary Fig. 4 shows the Gaussian fittings of the 4-CP QBS direct detachment-subtracted spectra in various control pulse intensity. This analysis gives better fit to the Gaussian fittings and broadening trends as the intensity of the control pulse increased.

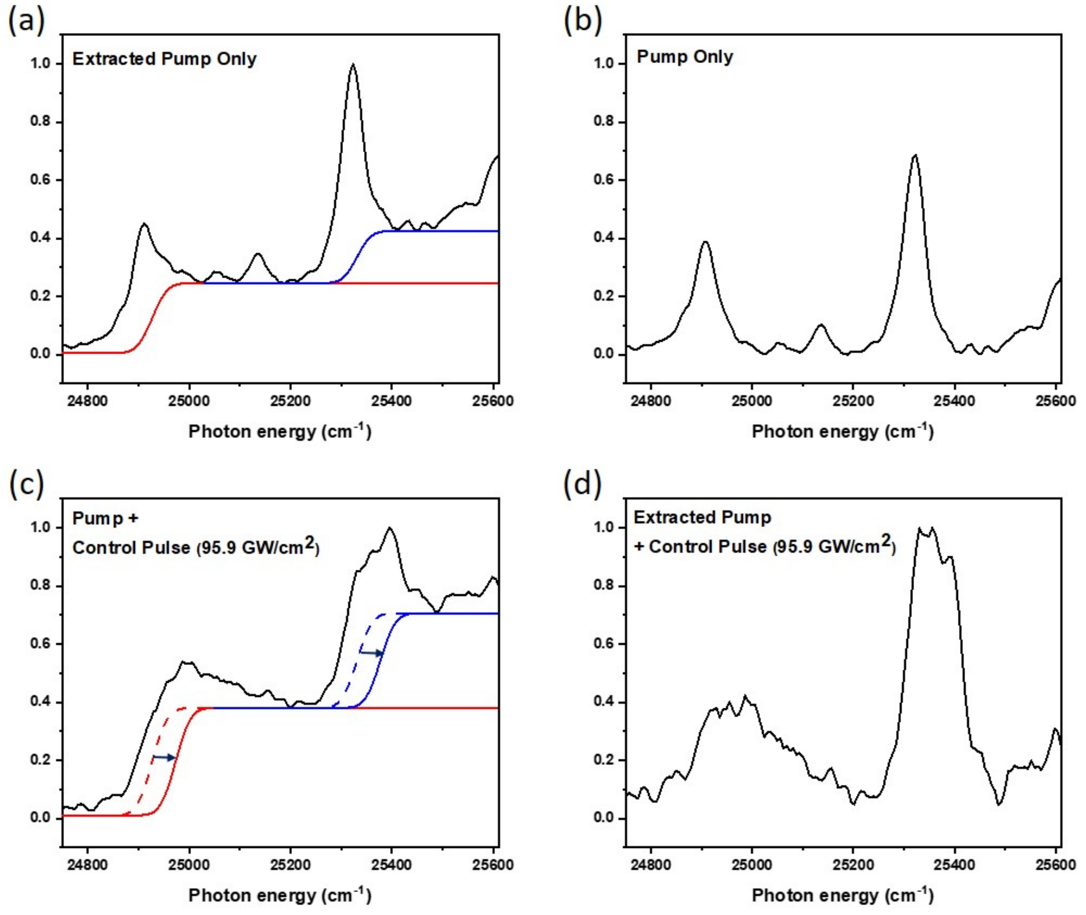

Supplementary Fig. 3 Subtraction procedures of the direct detachment features of 4-CP$^-$ QBS photodetachment spectra. (a) Pump only spectrum and error functions representing the stepwise feature at (red) EA and (blue) the most FC active $12'^1$ mode, respectively. (b) Resultant vibrational structures of the 4-CP$^-$ QBS. (c) Pump + control pulse (at 95.9 GW/cm^2) photodetachment spectrum and error functions to be subtracted. Note that the positions of the error functions were blue-shifted as the amount of the full (free-electron)-ponderomotive shift. (d) Resultant vibrational structures of the 4-CP QBS in the presence of the strong control pulse. Notably, as the vibrational Feshbach resonance bands quite stand out compared to the direct-detachment background electron signal, the observed ponderomotive shift in the 0 – 30 cm^{-1} range is little influenced by the subtraction of the direct-detachment within the error limit of ± 2 cm^{-1}.

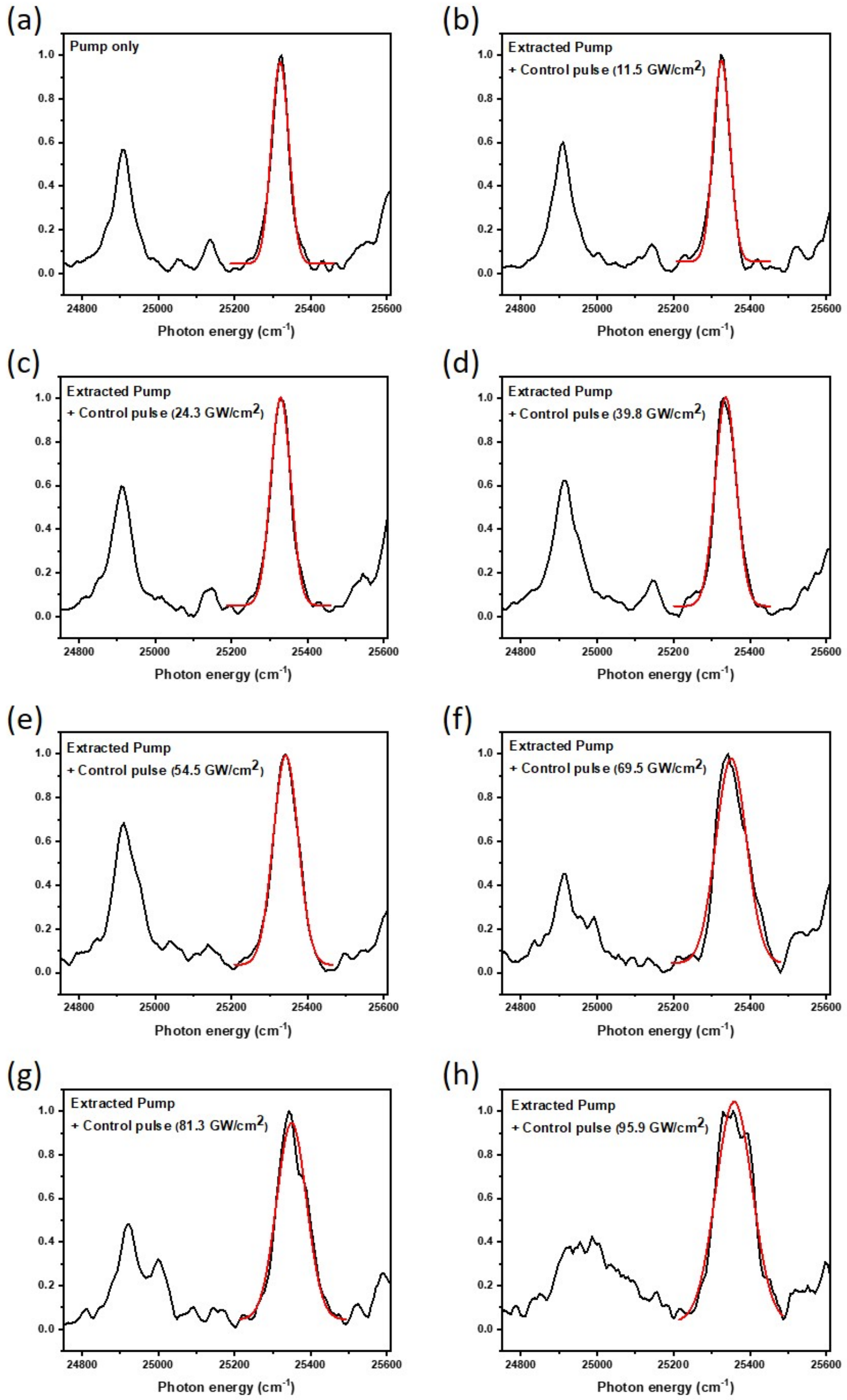

Supplementary Fig. 4 Stepwise direct detachment feature-subtracted spectra of 4-CP QBS and their Gaussian fitting at (a) pump only (without control pulse) and (b-h) with control pulse in various intensities.

Supplementary Note 2

Measurement of the laser intensity

In order to evaluate the laser intensity on the laser-ion interaction region, we measured laser power before and after the picosecond pulse passes through a couple of the CaF_2 windows. From the measured power differences, we could calculate the laser power at the laser-ion interaction region by considering the transmittance of a couple of CaF_2 windows.

$$P = P_{front} \times \sqrt{\frac{P_{back}}{P_{front}}} \qquad \text{Eq. S2}$$

Beam waist at the laser-ion interaction region was calculated using following equation;

$$\omega(z) = \omega_0 \sqrt{1 + \left(\frac{z\lambda}{\pi\omega_0^2}\right)^2} \qquad \text{Eq. S3}$$

, where z is the position of the laser-ion interaction region from the focus, ω_0 is the initial beam waist, and λ is the wavelength of the laser. Because of the low number density of the anion in the ion packet, we loosely focused control laser pulse away from the focal point. The peak power density of each laser shot was calculated by assuming the 1.7 ps pulse width Gaussian beam with 1 kHz repetition rates at $1/e^2$ (~13.5 % of peak) beam diameter.

$$P_{1/e^2} = \frac{1}{2}\pi I_0 \omega_0^2 (1 - e^2) \qquad \text{Eq. S4}$$

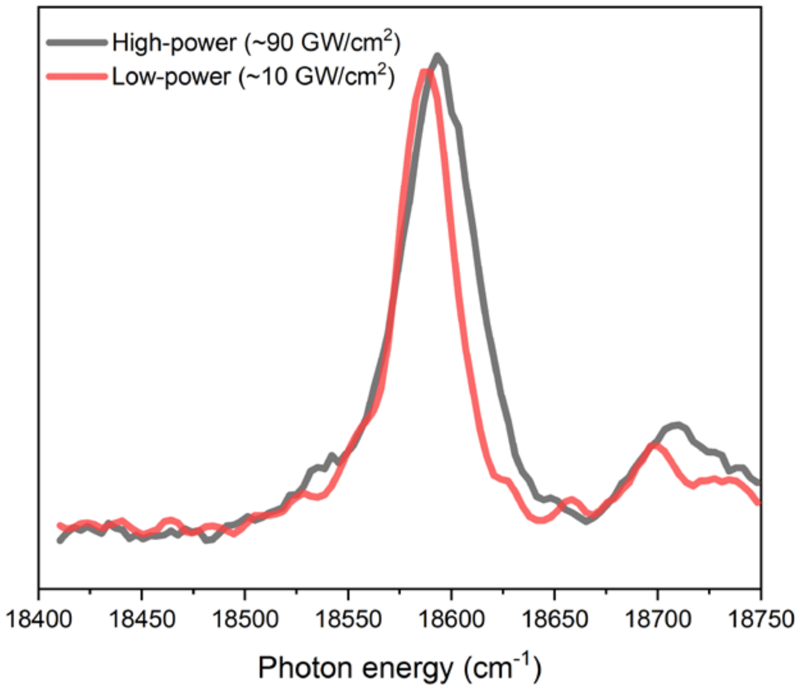

Supplementary Fig. 5 Picosecond pump pulse-only photodetachment spectra of the PhO^- DBS at low intensity (~10 GW/cm^2, red) and high intensity (~90 GW/cm^2, black) near the $11'^1$ resonant peak. Source data are provided as a Source Data file.

.

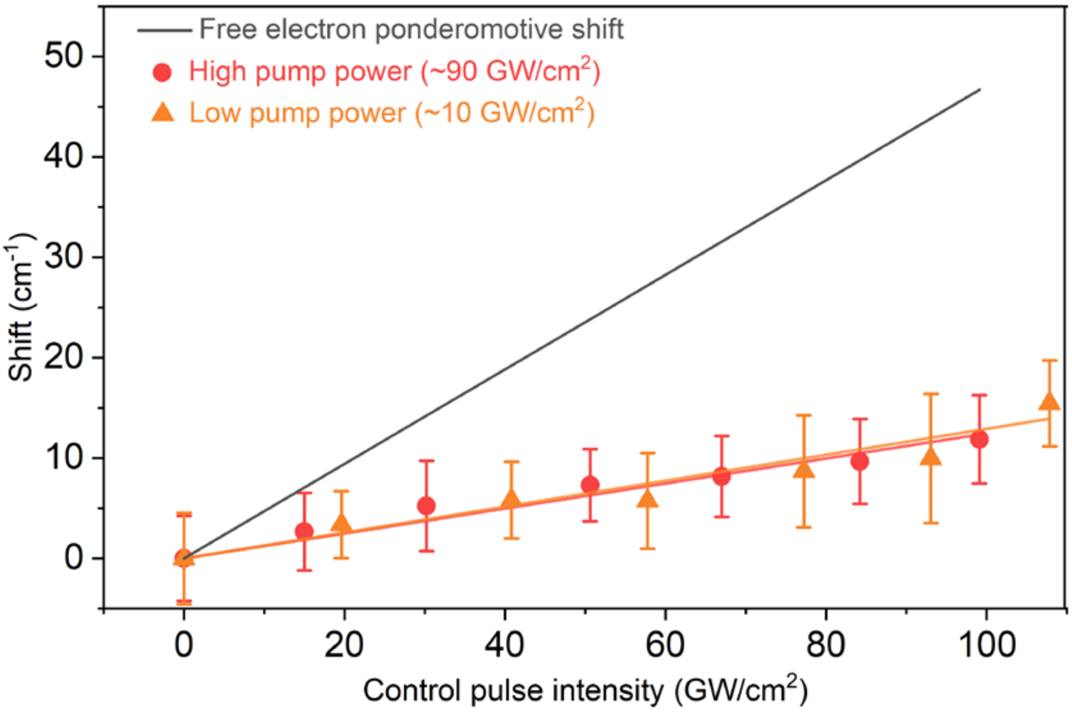

Supplementary Fig. 6 Ponderomotive shifts of the 11′[1] peak of the PhO⁻ DBS are plotted versus the intensity of the ps control laser pulse at high pump intensity (~90 GW/cm², red) and low pump intensity (~10 GW/cm², orange). The slope of the low pump intensity trend line was caluclated to be (0.274 ± 0.06) η, which is within the error bar obtained from the high pump intensity, (0.260 ± 0.05) η. The error bars represents the experimental errors (± σ) determined from the multiple measurements (n > 10) of the photodetachment spectrum. Source data at low pump power are provided as a Source Data file.

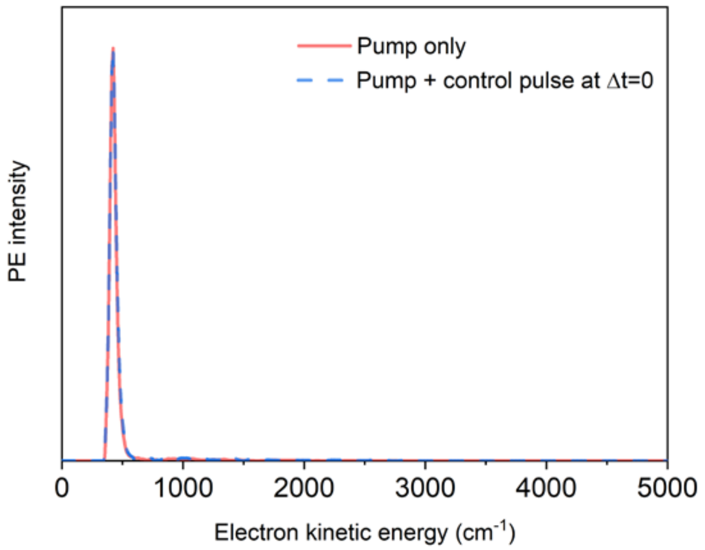

Supplementary Fig. 7 Resonant photoelectron spectra of the $11'^1$ PhO^- DBS with (blue dashed line) or without (red solid line) the control pulse (~90 GW/cm^2). They showed barely shifted spectral features, indicating that the autodetached electron with several ps lifetime barely affected by the ponderomotive shift of the both DBS resonant states and detachment threshold. Source data are provided as a Source Data file.

Supplementary References

1. Zhu, G.-Z., Qian, C.-H., Wang, L.-S. Dipole-bound excited states and resonant photoelectron imaging of phenoxide and thiophenoxide anions. *J. Chem. Phys.* **149**, 164301 (2018).
2. Zhu, G.-Z., Wang, L.-S. High-resolution photoelectron imaging and resonant photoelectron spectroscopy via noncovalently bound excited states of cryogenically cooled anions. *Chem. Sci.* **10**, 9409-9423 (2019).
